# The effectiveness of a 10-week family-focused e-Health healthy lifestyle program for school-aged children with overweight or obesity: a randomised control trial

**DOI:** 10.1186/s12889-024-21120-5

**Published:** 2025-01-07

**Authors:** Diana Zhu, Aimee L. Dordevic, Simone Gibson, Zoe E. Davidson

**Affiliations:** 1 Department of Nutrition Dietetics and Food, Monash University, Level 1, 264 Ferntree Gully Road, Notting Hill, VIC 3168 Australia; 2 School of Clinical Sciences, Monash University, Level 5 Block E, Monash Medical Centre, Clayton, VIC 3168 Australia; 3 Monash Centre for Scholarship in Health Education, Monash University, 27 Rainforest Walk, Clayton, Vic 3168 Australia

**Keywords:** E-health, Obesity, Web-based, Family-based

## Abstract

**Background:**

Electronic health (e-Health) interventions may increase effectiveness and address limitations of conventional in-person childhood obesity treatment programs. This study evaluates the effectiveness of a 10-week e-Health (web-based) healthy lifestyle program for school-aged children with overweight/obesity.

**Methods:**

In this randomised control trial (RCT), families with children aged 7–13 years with overweight/obesity (body mass index, BMI ≥ 85th percentile), living in Victoria, Australia, were recruited. Families were randomised to intervention or waitlist control and received the 10-week web-based program. The primary outcome was the change in children’s BMI z-score over 10 weeks. Other outcomes included change in children’s waist circumference, dietary intake, physical activity, and quality of life over 10 weeks.

**Results:**

Of 148 children (125 families) recruited, 102 children (85 families) completed the RCT. A significant decrease in children’s BMI z-scores was observed in the intervention compared to the control group over 10 weeks (mean difference 0.11; 95% CI, 0.02–0.20). The web-based program was also effective in improving children’s quality of life and lifestyle behaviour changes over 10 weeks. In the intervention group, there was a significant increase in children’s quality of life scores (intervention versus control: median change (IQR) = 11 (3, 17) versus 1 (-3, 7); *p* = 0.034), overall diet quality scores (6 (2, 10) versus 2 (-3, 5); *p* < 0.001), and daily physical activity levels (5.2 (-2.6, 12.8) versus − 0.2 (-8.2, 4.5); *p* = 0.022) compared to the control group.

**Conclusions:**

A web-based healthy lifestyle program effectively improved short-term health-related outcomes in children with overweight/obesity. Further research is needed to identify determinants of program effectiveness, evaluate sustained effects, and equitably tailor childhood obesity e-Health interventions to diverse populations.

**Trial registration:**

This study was registered with the Australian New Zealand Clinical Trial Registry (ACTRN12621001762842) on November 11, 2021, https://www.anzctr.org.au/Trial/Registration/TrialReview.aspx?id=383053.

**Supplementary Information:**

The online version contains supplementary material available at 10.1186/s12889-024-21120-5.

## Background

 Childhood obesity is a global issue, with the prevalence of children living with severe obesity (i.e. body mass index (BMI) ≥ 99.8 percentile), steadily increasing since the 20th century [1–5]. In developed countries (e.g. European countries, USA, Australia), at least 5% of school-aged children live with obesity, of which 1 in 4 of children live with severe obesity [1–3]. Childhood obesity has short- and long-term health implications, including adversely affecting growth, development (physical, emotional, psychosocial), and influencing the risk of other high-cost health conditions [6–8]. Younger children with overweight/obesity are also more likely to continue living with overweight/obesity into adulthood [8].

Unprecedented high childhood obesity rates relate in part to children living in environments that encourage the learning and adaptation of unhealthy lifestyle behaviours, including consuming a diet comprised predominantly of energy-dense/non-core foods and replacing activity with sedentary behaviours [7]. Globally, less than 50% of school-aged children meet recommendations for health-supporting lifestyle behaviours, such as physical activity [9]. In Australia, about 1 in 10 school-aged children or less meet population recommendations for physical activity and nutrition [10, 11].

Multicomponent, family-based healthy behaviour/lifestyle interventions are beneficial and recommended for the treatment of childhood overweight and obesity [6, 12–18]. However, treatment services are largely unavailable or inaccessible to many families [12, 14, 18, 19]. Treatment services are often delivered in-person in clinical/specialty settings in metropolitan areas, with strict eligibility criteria (e.g. children with co-morbidities) or have limited enrolment, and require an out-of-pocket fee [12, 14, 18, 19]. Such features limit the reach and scalability of conventional treatment services for childhood obesity [12–15].

With the ubiquitous and avid use of the internet, including by families residing in areas with high socio-economic disadvantage or remote locations, using digital technologies to transform conventional childhood obesity treatment services via electronic health (e-Health) interventions, has the potential to address limitations of effective childhood obesity treatment interventions delivered in-person [20]. Specifically, e-Health interventions have the potential to overcome these issues of demand, reach, and scalability. Studies have found that e-Health interventions, with the digital component serving as the adjunct or predominant part of the intervention, have improved the accessibility, efficiency, and reach of healthcare services [6, 13–15, 21–23]. E-Health childhood overweight/obesity treatment programs may also serve as favourable alternatives to existing services delivered face-to-face, for families needing treatment. E-Health interventions have characteristics that may appeal to or agree with families, including children and their ways of living [13, 15, 24]. Children actively and regularly engage with screen-based activities, including to access health-related information [10, 13, 15, 24]. Therefore, children may prefer to receive health-related services through e-Health modalities.

Research evaluating the effectiveness of e-Health interventions for the treatment of childhood overweight and obesity, have found that such treatment programs have improved weight-based and adiposity-related outcomes and/or lifestyle behaviours and habits in school-aged children, with varying effect sizes (negligible to at least small and significant effects) [13, 25]. Limited evidence-based, family-focused e-Health interventions for treating childhood overweight/obesity are available to meet the growing number of families with children needing treatment. More research is needed to adapt the e-Health interventions to childhood obesity treatment, namely studies on the effectiveness of family-focused e-Health treatment interventions, with key treatment components delivered through e-Health technologies and through a mode of digital technology (e.g. web-based treatment programs), and involving education that minimises contact time with health professionals.

This study evaluates the effectiveness of one such e-Health intervention, the online Better Health Program (BHPO) – a web-based healthy lifestyle program with health coaching sessions for families, adapted from the in-person group-based version (Better Health Program) (ACTRN12621001762842).The Better Health Program is based on evidence-based healthy lifestyle behaviour guidelines in Australia and has been found to improve weight-based outcomes in participating children post-program completion [26]. Key program content and duration of BHPO are identical to the Better Health Program. BHPO will be referred to hereon as web-based program.

## Methods

### Study design and recruitment

The study received ethics approval from Monash University Human Research Ethics Committee (30472) and the study protocol has been described in detail elsewhere [26]. Briefly, the RCT included a waitlisted control group, collected all data online, and delivered the web-based program as it would usually run outside of the study. This manuscript of the study is reported in line with the Consolidated Standards of Reporting Trials (CONSORT) statement [27].

Eligible children were aged 7–13 years with overweight or obesity, as defined by the Centers for Disease Control and Prevention (CDC) (BMI ≥ 85th percentile), living in Victoria, Australia, and accompanied by at least one parent/caregiver (referred to hereon as families), were recruited [28]. Families needed to be able to commit to completing weekly online modules and attending weekly coaching appointments scheduled from 15:00–21:00 on weekdays. Exclusion criteria were: families being unable to understand English or access a digital device to complete program activities, children requiring medical clearance for a health condition, were engaging or would be engaging in other weight management programs prior to the completion of the web-based program, or had a sibling who was recruited during an earlier recruitment cycle.

The administrating organisation Better Health Company (BHCo), a private company that collaborates with government and community organisations to deliver evidence-based healthcare services, recruited families from their established networks (e.g. general practitioners, other health professionals, schools, local government councils) using various promotion methods (e.g. school newsletters, flyers, posters, social media posts and campaigns) [26]. Recruitment was also promoted through professional clinical networks of the research team. Families were recruited from April 2022 to July 2023, and allocated (1:1 ratio) to the Intervention or Control group, via Research Randomizer (https://www.randomizer.org/). Families in the Intervention group received the web-based program in the next commencing school term/midterm. Families in the Control group were waitlisted for a control period of 10 weeks before receiving the web-based program. During the control period, families did not receive any information or education. Sample size was estimated a-priori using pilot data from the administrating organisation. To detect a difference in the mean change in BMI z-score units (Cohen’s d = 0.6, medium effect size) at 80% power and with a 30% attrition rate, 59 participants per group were required [26].

### Intervention

The 10-week web-based program is an evidence-based family-focused lifestyle program, that includes key content and behaviour change strategies from the group-based version, consistent with Australian guidelines on health-supporting lifestyle behaviours (healthy eating and physical activity). During the 10 weeks, families accessed weekly modules through a password secured website, with modules on healthy eating, physical activity and family-centred behaviour changes. Families also had weekly phone calls with a health professional coach (coaches were dietitians, nutritionists, exercise physiologists), during which families discussed the week’s modules and coaches confirmed the completion of activities [26]. Each week, families engaged in activities to apply their learnings, including completing online quizzes and setting and achieving goals related to health-supporting lifestyle behaviours. The administrating organisation, ran the web-based program ten times during the trial; during seven Australian primary school terms (from Term 2 2022-Term 4 2023) and three midterm schedules following Term 1 2023.

### Outcome measures

Data for the RCT were collected based on the protocol as described previously [26]. In short, families self-reported outcome measures through online surveys at baseline and post-program completion (10 weeks). Sociodemographic information (e.g. contact details, including home/mailing addresses) was self-reported by parents/caregivers at initial screening and included in the participating children’s profile in BHCo’s data system. Postcodes were used to determine the socio-economic advantage of areas where the web-based program was accessed, using the Socio-Economic Indexes for Areas - Index of Relative Socio-Economic Advantage and Disadvantage (SEIFA - IRSAD) scores/deciles (higher scores suggesting more advantaged areas compared to areas with lower scores; decile 1: lowest 10% of scores of most disadvantaged areas; decile 10: highest 10% of scores of most advantaged areas) [29]. Sociodemographic information also included children’s ethnicity and parent/caregiver characteristics (e.g. mother or father, marital status, employment status, ownership of accommodation). Sociodemographic characteristics, along with children’s anthropometric (children’s weight, height, waist circumference) and quality of life (questions from the PedsQL 4.0 Generic Core Scales – child and parent reports) measures, were also collected using Research Electronic Data Capture (REDCap) [30, 31]. Families were provided with a measurement kit and instructions to take children’s anthropometric measurements (weight, height, waist circumference) [26]. Children’s physical activity and dietary intake measures were collected through validated questionnaires, the Youth Activity Profile (YAP) and Australian Eating Survey (AES) respectively [32, 33].

### Primary outcome measure

The primary outcome was change in BMI z-score from baseline to 10 weeks. Children’s standing weight and height measurements – self-reported by parents/caregivers using provided measurement kits and instructions on taking measurements, at each time point – were used to calculate BMI. Families used their own scales to measure children’s weight or reported weight taken with their general practitioner. Weight, height, and BMI z-scores were determined using CDC’s growth references [28].

### Secondary outcome measures

Secondary outcome measures included children’s waist circumference, dietary intake, physical activity, and quality of life. Children’s waist circumferences were also self-reported by parents/caregivers using the provided measurement kits and instructions. Children, supported by their parents/caregivers (i.e. parents clarified questions or completed the surveys with their children if needed), responded to questions on their dietary intake, physical activity, and quality of life. Information collected on dietary intake included total energy intake, macro/micronutrient intake, core- or non-core food intake, and diet quality (AES calculated scores – Australian Recommended Food Score (ARFS), including a score for total diet quality and core food groups) [34, 35]. The YAP included questions on children’s activity level at home and school, and calculated the amount of physical activity and sedentary behaviour times children engaged in per day (weekday, weekend, or on average) or week [32]. The scoring methods of the PedsQL were used to generate scores (0–100) on children’s own or parent’s perceptions of children’s overall quality of life and physical, emotional, social, and school functioning, with higher scores suggesting higher quality of life [31].

### Statistical analysis

IBM SPSS Statistics version 25 (2017, Amronk, NY) was used to conduct all statistical analysis. *P*-values < 0.05 were considered significant. Only data from families who completed the RCT (families who completed at least primary outcome assessments at 10-weeks) were included in the main analyses. Normality of the data was assessed by examining skewness and kurtosis values and results for the Shapiro-Wilk test. Descriptive statistics and independent samples t-test were used to examine children’s sociodemographic characteristics, including children’s age, participating parent/caregiver (mother or father) and socio-economic data, and outcome measures at baseline in Intervention compared to Control group. Independent samples t-test was used for the primary analysis comparing the difference in the mean change in BMI z-scores between groups over 10 weeks. Mixed analysis of variance (ANOVA) was also conducted to examine the impact of the intervention on BMI z-score over 10 weeks in Intervention versus Control group. Based on the normality of the data, independent samples t-tests or Mann-Whitney U tests were conducted to compare differences in the change in secondary outcome measures between groups from baseline to 10 weeks.

To assess the impact of the program with greater certainty, outcomes from families in the Control group following program completion (i.e. end of week 10 of web-based program) were examined. Mixed ANOVA was used to examine the impact of the web-based program in the Intervention versus Control group following program completion on BMI z-score. Within group analyses were also conducted on secondary outcome measures in the Control group immediately pre- to post-program completion.

To address attrition bias, an intention-to-treat analysis was performed on the primary outcome [36]. For participants with missing data at 10 weeks, baseline values were carried forward. Statistical analyses were performed on all participants as described above.

In accordance with the study protocol, outcome measures were repeated with families up to 12 months post-program completion. Data collection at 6- and 12-months post-intervention is ongoing and will be reported separately, following completion of all follow-up timepoints.

## Results

### Baseline characteristics

**Fig. 1:**
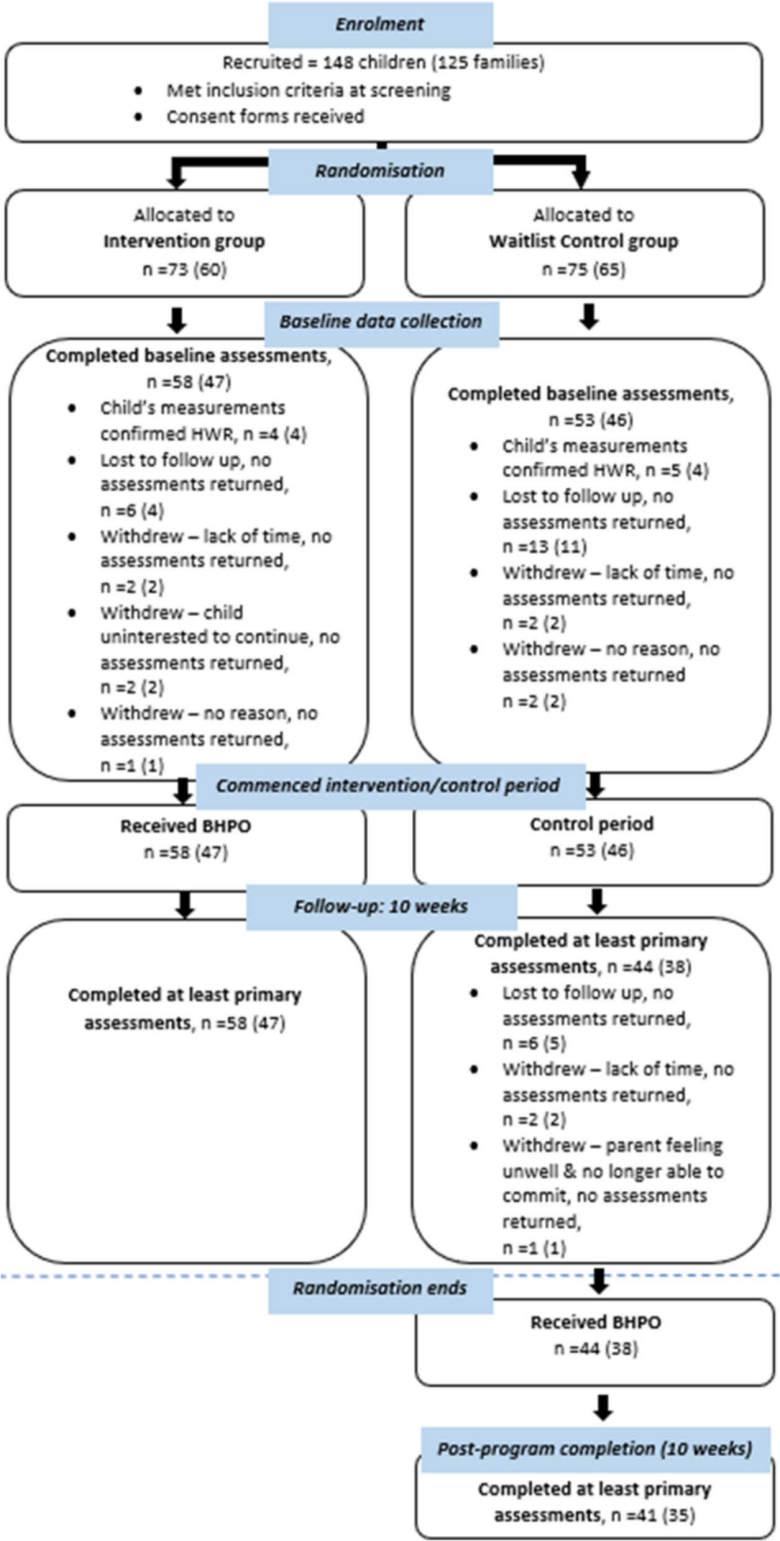
CONSORT flow diagram summarising flow of recruited participants from allocation to 10 weeks/post-program completion. Sample sizes reported as *n* = children (*n* = families); Abbreviations: HWR, healthy weight range; BHPO, Online Better Health Program; Child’s measurements confirmed HWR = child does not meet eligibility criteria

A total of 148 children (Intervention – 73, Control – 75) from 125 families were recruited to the study. Of the 73 children (60 families) who were randomised to the Intervention group, 58 children (47 families) completed baseline assessments and also completed at least the primary outcome measure following the web-based program (completed the RCT; 10 weeks). Of the 75 children (65 families) who were randomised to the Control group, 53 children (46 families) completed baseline assessments, and 44 children (38 families) completed at least the primary outcome measure at 10 weeks (completed the RCT; end of waitlist control period) and prior to receiving the web-based program. A CONSORT flow diagram of recruited participants is shown in Fig. 1.

Baseline characteristics of completers are summarised in Table 1 and S1. Supplementary tables (Table S1-S3), found in Additional Files 1 to 3, provide additional information on outcomes related to children’s dietary intake and physical activity. Sociodemographic characteristics were similar between groups. Participating children (49% girls) were on average aged 10.0 (1.9) years, living with obesity, and mostly accompanied by their mothers (93% children; 22% children in single-parent household). Sixty-five children (64%) identified as Australian and the remaining 36% identified as various other ethnicities, including Aboriginal Australian, New Zealander, Maori Pacific Islanders, South American, African, European, Asian, and Middle Eastern. Parents/primary caregivers mostly reported owning their own accommodation (75%) and were employed (83%). The web-based program was mostly accessed by children living in areas regarded as having higher socio-economic advantage (28% in most advantaged (deciles 1,2) areas; 14% in most disadvantaged (deciles 9,10) areas). Groups differed in parents’ perceptions of their children’s quality of life. Parents in the Intervention group gave lower scores for their children’s quality of life, including the emotional, social, and school functioning dimensions, compared with the Control group. Families in the Intervention group also reported that participating children consumed more fruit than Control group (*p* = 0.011).

**Table 1:** Baseline characteristics of completers (participating children)

Characteristic	Intervention (*n* = 58)	Control (*n* = 44)	*P*-value ^g^
**Age (y)**,** M (SD)**	10.2 (1.9)	9.7 (1.8)	0.268
**Gender**,** n (%)**^**a**^			0.567
Male	31 (53)	21 (48)	
Female	27 (47)	23 (52)	
**Parent**,** n (%)**^**a**^			1.000
Mother	54 (93)	41 (93)	
Father	4 (7)	3 (7)	
Single parent	16 (28)	7 (16)	0.162
**BMI (kg/m**^**2**^**)**,** M (SD)**	25.4 (4.2)	25.5 (5.4)	0.848
Weight (kg), M (SD)	57.1 (18.9)	53.5 (17.3)	0.324
Height (cm), M (SD)	148.0 (15.2)	143.2 (11.6)	0.087
**BMI z-score**,** M (SD)**	1.95 (0.42)	1.96 (0.50)	0.861
Weight z-score, M (SD)	2.10 (0.75)	2.05 (0.72)	0.742
Height z-score, M (SD)	1.24 (1.20)	0.95 (1.25)	0.246
**Waist circumference (cm)**,** M (SD)**^**c**^	79.6(19.8)	78.4 (19.3)	0.766
**Dietary intake**,** Md (IQR)**^**b, d**^			
Total energy intake (kJ/d)	9998 (8247, 11275)	9359 (6945, 11116)	0.158
Macronutrient intake			
Carbohydrate (%EI)	48 (44, 51)	46 (43, 50)	0.208
Protein (%EI)	16 (14, 17)	17 (14, 19)	0.139
Fat (%EI)	36 (34, 39)	35 (33, 38)	0.833
Saturated fat (%EI)	14 (13, 16)	15 (14, 16)	0.681
Sugars (g/d)	124 (95, 157)	119 (85, 148)	0.503
Fibre (g/d)	27 (22, 32)	26 (22, 32)	0.900
Sodium (mg/d)	2474 (1959, 2931)	2192 (1627, 2827)	0.121
Nutrient-dense/core foods (%EI)	56 (49, 64)	65 (49, 69)	0.100
Energy-dense/non-core foods (%EI)	44 (36, 51)	36 (31, 52)	0.100
Total diet quality (ARFS 0–73)	29 (24, 34)	29 (24, 38)	0.644
**Physical activity**,** Md (IQR)**^**b, e**^			
Overall average PA time (min/d)	72.4 (50.5, 89.8)	62.9 (51.8, 92.9)	0.977
Overall average PA time (min/wk)	506.5 (353.3, 628.4)	440.4 (362.4, 650.5)	0.977
Sedentary behaviour			
Average total sedentary time (min/d)	3.8 (3.3, 4.1)	3.8 (3.3, 3.9)	0.455
Average total sedentary time (min/wk)	19.0 (16.5, 20.4)	18.8 (16.3, 19.6)	0.455
**Quality of life – parent report**,** Md (IQR)**^**b, f**^			
Overall quality of life	66 (51, 74)	76 (56, 84)	0.005
Physical functioning	69 (49, 84)	77 (55, 90)	0.148
Emotional functioning	55 (44, 65)	65 (50, 80)	0.004
Social functioning	60 (45, 75)	80 (60, 90)	0.002
School functioning	70 (55, 80)	75 (60, 90)	0.020
**Quality of life – child report**,** Md (IQR)**^**b, f**^			
Overall quality of life	66 (53, 76)	71 (57, 85)	0.095
Physical functioning	73 (56, 85)	80 (63, 91)	0.079
Emotional functioning	55 (45, 70)	60 (45, 78)	0.472
Social functioning	65 (50, 81)	78 (55, 90)	0.110
School functioning	70 (55, 80)	75 (56, 89)	0.272

*M* mean; *Md* median; *y* years; *d* day; *wk* week; *kJ* kilojoules; *%EI* percentage of energy intake; *ARFS* Australian recommended food score; *PA* physical activity; *min* minutes

^a^Chi-Square Test was conducted to test for associations in outcomes between groups

^b^Mann-Whitney U Test was conducted to test for differences in outcomes between groups

^c^57/58 children completed waist circumference in the Intervention group

^d^42/44 children completed Australian Eating Survey in the Control group

^e^41/44 children completed Youth Activity Profile in the Control group

^f^Quality of life scores: 0–100; scores closer to 100 suggesting higher quality of life

^g^Independent samples t-test was conducted to test for differences in outcomes between groups

### Compliance

Key components of the web-based program included the weekly online modules and phone coaching sessions. Web-based modules were marked complete in the administrating organisation’s program database when families attempted at least 75% of the weekly activities. During phone coaching calls, coaches recorded attendance of the coaching session and confirmed families’ engagement with the web-based modules, including whether they completed activities on health-supporting lifestyle behaviour changes. Families on average completed 9/10 web-based modules and 9/10 health coaching sessions. All families engaged (marked complete for web-based and/or coaching session) with at least 50% of the web-based program. 88% of children (89% of families) from the Intervention group and 89% of children (87% of families) from the Control group completed 10/10 online sessions and web-based activities. 90% of children (89% of families) from the Intervention group and 86% of children (84% of families) from the Control group completed 10/10 coaching sessions.

### Change in outcome measures over 10 weeks

Tables 2 and 3, S2, and S3 summarise results from analyses comparing the differences in outcome measures between groups from baseline to 10 weeks.

**Table 2:** Between group differences in change in anthropometric outcome measures from baseline to 10 weeks

Characteristic M (SD)	Intervention (*n* = 58)			Control (*n* = 44)			Between group difference		
	Baseline	10 weeks	Change	Baseline	10 weeks	Change	MD (95% CI)	*P*-value ^b^	Cohen’s d
**BMI (kg/m**^**2**^**)**	25.4 (4.2)	24.8 (4.4)	−0.6 (1.2)	25.5 (5.4)	25.6 (5.5)	0.1 (1.4)	0.7 (0.1, 1.2)	0.015	0.5
Weight (kg)	57.1 (18.9)	57.0 (19.1)	−0.0 (1.9)	53.5 (17.3)	54.3 (17.5)	0.9 (2.8)	0.9 (−0.0, 1.8)	0.057	0.4
Height (cm)	148.0 (15.2)	149.5 (14.9)	1.6 (2.1)	143.2 (11.6)	144.2 (11.8)	1.0 (1.9)	−0.6 (−1.4, 0.2)	0.136	−0.3
**BMI z-score**	1.95 (0.42)	1.81 (0.54)	−0.13 (0.23)	1.96 (0.50)	1.93 (0.50)	−0.03 (0.23)	0.11 (0.02, 0.20)	0.018	0.5
Weight z-score	2.10 (0.75)	1.97 (0.95)	−0.13 (0.26)	2.05 (0.72)	2.01 (0.74)	−0.04 (0.17)	0.09 (0.00, 0.18)	0.052	0.4
Height z-score	1.24 (1.20)	1.27 (1.22)	0.03 (0.29)	0.95 (1.25)	0.91 (1.26)	−0.05 (0.29)	−0.08 (−0.20, 0.03)	0.161	−0.3
**Waist circumference (cm)**^**a**^	79.6 (19.8)	76.2 (19.9)	−2.2 (19.2)	78.4 (19.3)	77.8 (18.6)	−1.0 (13.6)	1.3 (−5.7, 8.2)	0.720	0.1

*M* mean; *SD* standard deviation; *MD* mean difference; *CI* confidence interval

^a^53/58 children completed waist circumference in the Intervention group; 43/44 children completed waist circumference in the Control group

^b^Independent samples t-test was conducted to test for differences in outcomes between groups

**Table 3:** Between group differences in change in secondary outcome measures from baseline to 10 weeks

Characteristic Md (IQR)	Intervention (*n* = 58)				Control (*n* = 44)				*P*-value^a^
	Baseline	10 weeks		Change	Baseline		10 weeks	Change	
**Dietary intake**^b^									
Total energy intake (kJ/d)	9998 (8247, 11275)	9062 (7898, 10699)		−1514 (−2639, 197)	9359 (6945, 11116)		9501 (7319, 11445)	14 (−1597, 1125)	0.010
Macronutrient intake									
Carbohydrate (%EI)	48 (47, 52)	47 (43, 50)		−1 (−4, 1)	47 (45, 51)		48 (44, 51)	0 (−4, 3)	0.314
Protein (%EI)	16 (14, 17)	18 (16, 20)		2 (1, 4)	17 (15, 19)		16 (15, 18)	0 (−2, 1)	< 0.001
Fats (%EI)	36 (33, 39)	35 (32, 37)		−1 (−4, 1)	36 (33, 39)		36 (34, 39)	0 (−2, 2)	0.070
Saturated fat (%EI)	14 (13, 16)	14 (12, 16)		−1 (−2, 1)	15 (14, 16)		15 (14, 17)	0 (−1, 1)	0.002
Sugars (g/d)	124 (95, 157)	113 (84, 143)		−11 (−42, 12)	119 (85, 148)		118 (79, 154)	−1 (−29, 24)	0.105
Fibre (g/d)	27 (22, 32)	28 (23, 34)		3 (−3, 5)	26 (22, 32)		26 (22, 34)	1 (−4, 6)	0.577
Sodium (mg/d)	2474 (1959, 2931)	2044 (1752, 2467)		−476 (−810, −20)	2192 (1627, 2826)		2398 (1824, 2687)	−34 (−389, 459)	< 0.001
Nutrient-dense/core foods (%EI)	56 (49, 64)	71 (64, 77)		12 (5, 23)	65 (49, 69)		63 (51, 68)	−2 (−6, 5)	< 0.001
Energy-dense/non-core foods (%EI)	44 (36, 51)	29 (23, 36)		−12 (−23, −5)	36 (31, 52)		37 (32, 49)	2 (−5, 6)	< 0.001
Total diet quality (ARFS 0–73)	29 (24, 34)	36 (29, 41)		6 (2, 10)	29 (24, 38)		30 (25, 38)	2 (−3, 5)	< 0.001
**Physical activity**^**c**^									
Overall average PA time (min/d)	72.4 (50.5, 89.8)	72.9 (63.0, 91.2)		5.2 (−2.6, 12.8)	62.9 (51.8, 92.9)		60.0 (50.3, 92.4)	−0.2 (−8.2, 4.5)	0.022
Overall average PA time (min/wk)	506.5 (353.3, 628.4)	510.6 (440.8, 638.4)	36.5 (−18.2, 89.9)		440.4 (362.4, 650.5)	419.9 (352.0, 646.6)		−1.4 (−57.4, 31.8)	0.022
Sedentary behaviour									
Average total sedentary time (min/d)	3.8 (3.3, 4.1)	3.6 (3.2, 3.9)	−0.1 (−0.2, 0.0)		3.8 (3.3, 3.9)	3.7 (3.3, 3.9)		0.0 (−0.1, 0.1)	0.004
Average total sedentary time (min/wk)	19.0 (16.5, 20.4)	18.2 (15.8, 19.6)	−0.5 (−0.9, 0.0)		18.8 (16.3, 19.6)	18.4 (16.3, 19.4)		0.0 (−0.6, 0.3)	0.004
**Quality of life – parent report**^**d, e**^									
Overall quality of life	66 (51, 74)	76 (62, 85)	11 (3, 17)		76 (56, 84)	73 (60, 85)		1 (−3, 7)	< 0.001
Physical functioning	69 (49, 84)	81 (63, 91)	6 (0, 25)		77 (55, 90)	75 (59, 91)		2 (−5, 9)	0.022
Emotional functioning	55 (44, 65)	70 (55, 81)	13 (4, 25)		65 (50, 80)	68 (50, 79)		0 (−5, 9)	< 0.001
Social functioning	60 (45, 75)	75 (59, 90)	15 (5, 25)		80 (60, 90)	78 (60, 89)		0 (−5, 10)	< 0.001
School functioning	70 (55, 80)	80 (65, 90)	10 (0, 16)		75 (60, 90)	75 (65, 89)		0 (−10, 10)	0.003
**Quality of life – child report**^**d, e**^									
Overall quality of life	66 (53, 76)	78 (65, 84)	7 (0, 14)		71 (57, 85)	76 (66, 86)		2 (−3, 10)	0.034
Physical functioning	73 (56, 85)	81 (71, 94)	6 (−3, 19)		80 (63, 91)	81 (69, 94)		0 (−6, 6)	0.015
Emotional functioning	55 (45, 70)	65 (50, 80)	5 (−6, 15)		60 (45, 78)	65 (50, 80)		0 (−5, 15)	0.863
Social functioning	65 (50, 81)	75 (60, 90)	8 (−5, 20)		78 (55, 90)	75 (65, 89)		5 (−5, 15)	0.434
School functioning	70 (55, 80)	80 (65, 90)	10 (0, 20)		75 (56, 89)	78 (65, 90)		0 (−4, 10)	0.034

*Md* median; *IQR* interquartile range; *d* day; *wk* week; *kJ* kilojoules; *%EI* percentage of energy intake; *ARFS* Australian recommended food score; *PA* physical activity; *min* minutes

^a^Mann-Whitney U Test was conducted to test for differences in change in outcomes between groups

^b^Baseline: 53/58 children completed Australian Eating Survey in the Intervention group, 42/44 children completed Australian Eating Survey in the Control group; 10 weeks: 53/58 children completed Australian Eating Survey in the Intervention group, 44/44 children completed Australian Eating Survey in the Control group; Change in outcome measures calculated for 53/58 children from the Intervention group, 42/44 children from the Control group

^c^54/58 children completed Youth Activity Profile in the Intervention group; 41/44 children completed Youth Activity Profile in the Control group

^d^54/58 children completed PedsQL survey in the Intervention group

^e^Quality of life scores: 0–100; scores closer to 100 suggesting higher quality of life

### Primary outcome measure

The mean difference (95% confidence interval (CI)) in change in BMI z-score between groups was 0.11 (0.02, 0.20), *p* = 0.018, with a greater decrease in BMI z-scores observed in the Intervention group (mean (standard deviation (SD) reduction) = 0.13 (0.23) BMI z-score units) compared to a negligible change in BMI z-scores in the Control group (mean (SD) reduction = 0.03 (0.23) BMI z-score units). The changes in BMI z-scores were related to weight maintenance achieved in the Intervention group (mean weight (SD) change = 0.0 (1.9) kg) compared to an increase in weight observed in the Control group (mean weight (SD) change = 0.9 (2.8) kg), and in the context of height growth in both groups, at 10 weeks.

**Fig. 2:**
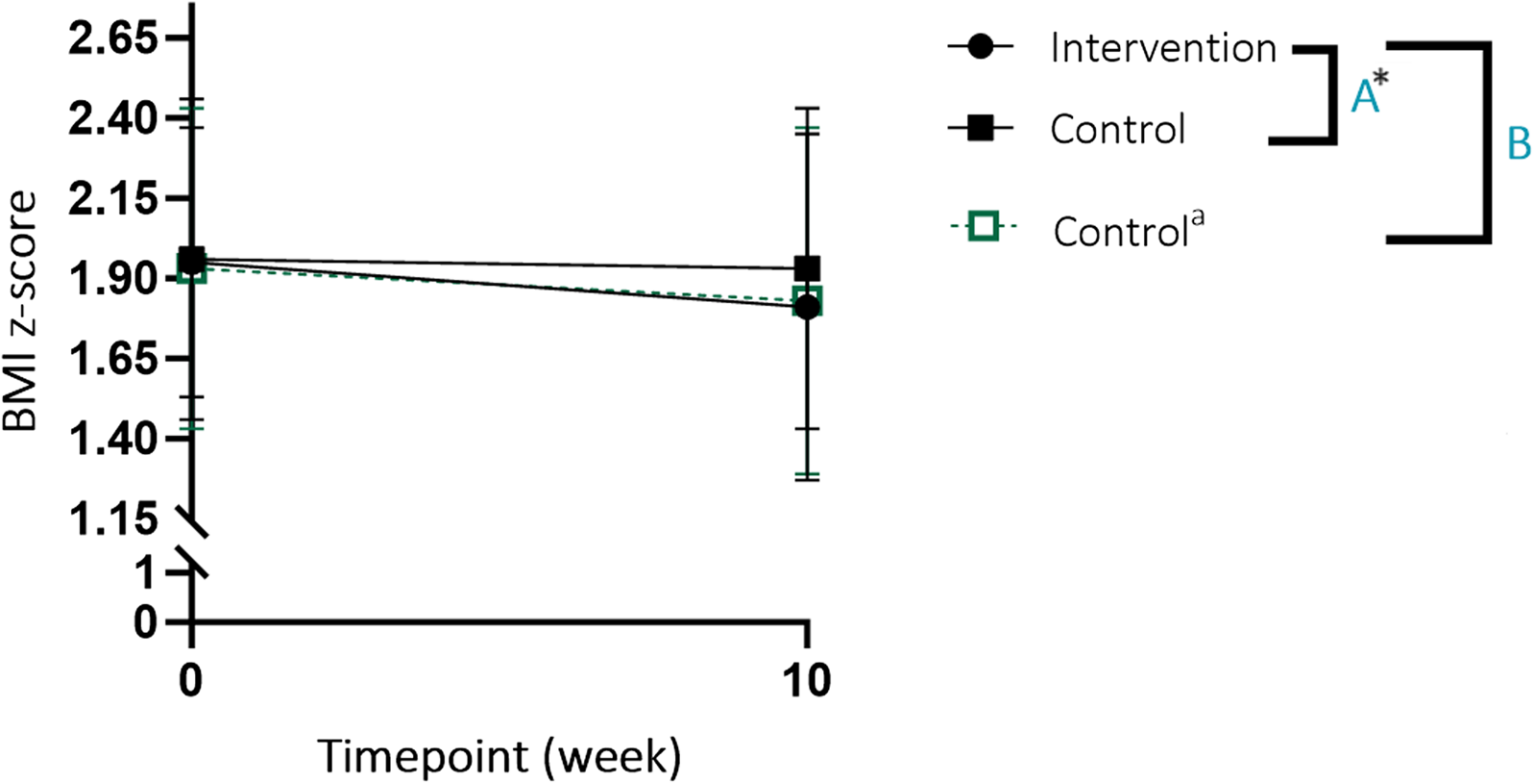
ANOVA results for change in BMI z-score over 10-weeks (following waitlist/program completion) between groups. ^a^Impact of web-based program on BMI z-score at immediately pre- to post-program completion in the Control group. ^*^Significant interaction between time and allocation (*p* = 0.018). **A** (Relationship A): the impact of web-based program compared to waitlist control on BMI z-score over 10-weeks. **B** (Relationship B): the impact of web-based program on BMI z-score over 10-weeks between groups

Mixed ANOVA analysis confirmed that the web-based program decreased BMI z-score over 10 weeks compared with a lack of change in BMI z-score with no intervention (waitlist control); an interaction was observed between time and allocation over 10 weeks (*p* = 0.018; Fig. 2, Relationship A).

Application of the intention-to-treat principles to analyse the change in children’s BMI z-score over 10 weeks, also resulted in a significant difference in the change in children’s BMI z-scores over 10 weeks between groups (mean difference (CI) = 0.10 (0.02–0.18), *p* = 0.016). Compared with the original analysis, a greater decrease was observed in children’s BMI z-scores in the Intervention (mean (SD) reduction = 0.13 (0.23) BMI z-score units) compared to the Control group (mean (SD) reduction = 0.03 (0.21) BMI z-score units).

### Secondary outcome measures

Compared to children in the Control group, children in the Intervention group had a greater and favourable changes in daily energy, saturated fat, and sodium intake, as well as intake of core and non-core foods, following the web-based program. Diet quality, as reflected by the ARFS, increased from a median score 29 (‘*needs work*’ category) to median score 36 (‘*getting there*’ category) in the Intervention group, whilst remaining unchanged in the Control group (‘*needs work*’ category). A greater increase in children’s engagement in physical activity and concurrent decrease in sedentary behaviours was observed in the Intervention group (median (interquartile range (IQR)) increase in daily average physical activity = 5.2 (−2.6, 12.8) minutes per day) compared to negligible changes in the Control group (*p* = 0.022).

Parents in the Intervention group reported a greater improvement in their children’s overall quality of life (median (IQR) change in overall quality of life score = 11 (3, 17)), and in all physical and psychosocial domains, compared to little/no change in the Control group (*p* < 0.001). Children in the Intervention group also reported a greater improvement in their own overall quality of life (median (IQR) change in overall quality of life score = 7 (0, 14)) compared to the Control group (*p* = 0.034), including in the physical and school functioning dimensions.

### Impact of the web-based program on outcome measures in the control group

Children in the Control group who completed at least the primary assessments at post-program completion were included in the within-group analyses of outcome measures (41 children/35 families). Analyses of outcome measures in the Control group, following program completion, found similar impact of the program as observed in the Intervention group (Table 4 and S3). A decrease in BMI z-score post-program completion (mean (SD) reduction of 0.13 (0.21) BMI z-score units), including weight maintenance, was observed (*p* < 0.001). Mixed ANOVA confirmed BMI z-score decreased after 10 weeks of the program, irrespective of the Control group undergoing 10 weeks waiting period prior to receiving the web-based program; no interaction effect was observed between time and allocation over the 10 weeks duration of the program (Fig. 2, Relationship B).

**Table 4:** Within group differences in outcome measures for the control group immediately pre-to post-program completion

Characteristic	Immediately pre-program/10 weeks (*n* = 44)	Immediately post-program completion (*n* = 44)	*P*-value ^a^
**BMI (kg/m**^**2**^**)**,** M (SD)**^**b**^	25.8 (5.6)	25.2 (6.0)	0.023
Weight (kg), M (SD) ^b^	54.6 (18.1)	54.6 (18.8)	0.993
Height (cm), M (SD) ^b^	144.1 (12.2)	145.7 (11.3)	< 0.001
**BMI z-score**,** M (SD)**^**b**^	1.95 (0.50)	1.83 (0.54)	< 0.001
Weight z-score, M (SD) ^b^	2.05 (0.73)	1.94 (0.74)	< 0.001
Height z-score, M (SD) ^b^	0.97 (1.22)	1.03 (1.25)	0.394
**Waist circumference (cm)**,** M (SD)**^**c**^	76.3 (18.2)	78.0 (14.5)	0.352
**Dietary intake**,** Md (IQR)**^**d**^			
Total energy intake (kJ/d)	9501 (7319, 11445)	9315 (7632, 11256)	0.748
Macronutrient intake			
Carbohydrate (%EI)	48 (44, 51)	46 (43, 50)	0.065
Protein (%EI)	16 (15, 18)	18 (16, 19)	< 0.001
Fats (%EI)	36 (34, 39)	35 (33, 38)	0.313
Saturated fat (%EI)	15 (14, 17)	14 (12, 16)	0.031
Sugars (g/d)	118 (79, 154)	107 (91, 137)	0.264
Fibre (g/d)	26 (22, 34)	29 (23, 38)	0.036
Sodium (mg/d)	2398 (1824, 2687)	2399 (1814, 2695)	0.460
Nutrient-dense/core foods (%EI)	63 (51, 68)	70 (62, 76)	< 0.001
Energy-dense/non-core foods (%EI)	37 (32, 49)	30 (24, 38)	< 0.001
Total diet quality (ARFS 0–73)	30 (25, 38)	40 (31, 45)	< 0.001
**Physical activity**,** Md (IQR)**^**e**^			
Overall average PA time (min/d)	60.0 (50.3, 92.4)	70.1 (56.7, 95.9)	< 0.001
Overall average PA time (min/wk)	419.9 (352.0, 646.6)	490.6 (397.2, 671.2)	< 0.001
Sedentary behaviour			
Average total sedentary time (min/d)	3.7 (3.3, 3.9)	3.7 (3.1, 3.8)	< 0.001
Average total sedentary time (min/wk)	18.4 (16.3, 19.4)	18.3 (15.7, 19.1)	< 0.001
**Quality of life – parent report**,** Md (IQR)**^**e, f**^			
Overall quality of life	73 (60, 85)	82 (57, 89)	0.199
Physical functioning	75 (59, 91)	88 (50, 96)	0.661
Emotional functioning	68 (50, 79)	75 (60, 90)	0.019
Social functioning	78 (60, 89)	83 (70, 99)	0.025
School functioning	75 (65, 89)	80 (60, 95)	0.108
**Quality of life – child report**,** Md (IQR)**^**d, f**^			
Overall quality of life	76 (66, 86)	83 (70, 93)	0.005
Physical functioning	81 (69, 94)	88 (72, 97)	0.230
Emotional functioning	65 (50, 80)	70 (50, 90)	0.074
Social functioning	75 (65, 89)	90 (70, 100)	0.034
School functioning	78 (65, 90)	80 (70, 95)	0.053

*M* mean; *Md* median; *IQR* interquartile range; *d* day; *wk* week; *kJ* kilojoules; *%I*, percentage of energy intake; *ARFS* Australian recommended food score; *PA* physical activity; min, minutes

^a^Wilcoxon Signed Rank test was conducted to test for changes in outcome measures from pre- to post-program completion

^b^Analyses conducted with data from 41/44 children

^c^Analyses conducted with data from 38/44 children

^d^Analyses conducted with data from 39/44 children

^e^Analyses conducted with data from 40/44 children

^f^Quality of life scores: 0–100; scores closer to 100 suggesting higher quality of life

Similar to the favourable changes in outcome measures as seen in the Intervention group, immediately post-program completion, children in the Control group consumed a higher percentage of energy intake from core foods and lower percentage of energy intake from non-core foods compared to pre-program commencement (median percent energy intake (IQR) from core foods increased from 63 (51, 68)% to 70 (62, 76)%, median percent energy intake (IQR) from non-core foods decreased from 37 (32, 49)%, to 30 (24, 38)%; *p* < 0.001). Children also engaged in more physical activity versus sedentary behaviour following the program, with physical activity increasing from median time (IQR) of 60.0 (50.3, 92.4) to 70.1 (56.7, 95.9) minutes per day (*p* < 0.001). A favourable change was observed in children’s quality of life, with children reporting a higher overall quality of life (median score (IQR) increased from 76 (65, 86) to 83 (70, 93)), notably in the physical (median score (IQR) increased from 73 (56, 85) to 81 (71, 94)) and school functioning (median score (IQR) increased from 70 (55, 80) to 80 (65, 90)) domains.

## Discussion

In this study, a family-focused web-based program effectively improved health-related outcome measures in children with overweight and obesity. Interventions for treating childhood obesity aim to achieve weight maintenance, in the context of height growth, by supporting children with adapting healthy lifestyle behaviours [6, 7, 18]. Following the web-based program, a significant, moderate effect on the change in children’s BMI was observed between groups, noting a wide variation in responses. Likewise, following program completion, a clinically significant mean (SD) reduction of 0.13 (0.23) BMI z-score units (weight maintenance and height growth) was observed in participating children. A decrease of at least 0.10 BMI z-score units in children aged 7–17 years, has been suggested to improve metabolic health outcomes (e.g. improved insulin sensitivity/resistance, lipid profile) [6, 37, 38]. The decrease in BMI z-score observed in this study is comparable to the reduction in BMI z-score reported in research on a similar in-person group-based version of the program [39, 40]. The change in BMI z-scores is also greater than the average decrease of 0.05 (95% CI, −0.10 to −0.01) BMI z-score units, following completion of healthy lifestyle programs, found in a Cochrane review on 20 RCT’s examining the effectiveness of family-based healthy lifestyle programs (including content on both healthy eating and physical activity) to similarly aged children (6–12 years) [41]. This finding suggests that e-Health healthy lifestyle programs can preserve, if not optimise, the impact of effective weight management programs delivered in-person and in specialty/community settings. The change in BMI z-scores found in this study is greater than the decrease in BMI z-scores achieved by children who completed other e-Health childhood obesity interventions. Based on findings from 13 studies examining the impact of e-Health interventions, a 2021 meta-analysis reported a mean decrease of 0.063 BMI z-score units achieved by participating children [13]. This may be related to the web-based program targeting the family unit, providing evidence-based content on healthy eating, physical activity and behaviour change strategies, with individualised support from health coaches, and high compliance achieved during the intervention period, however requires further exploration.

The web-based program effectively influenced healthy behaviour changes, including improving participating children’s dietary intake and physical activity levels, in both groups post-program completion. At 10 weeks, significant differences were found between cohorts regarding changes to daily energy, sodium, core food and non-core food intake, diet quality, and physical activity behaviours. Likened to other e-Health interventions and a parallel group-based program, the web-based program influenced children to increase their intake of core foods (e.g. vegetables) and decrease their intake of non-core foods (e.g. discretionary foods, energy-dense take-away meals), including similar change in percentage energy from core foods and non-core foods, increase their physical activity levels, and decrease sedentary behaviours [23, 40, 42]. Findings suggest that the web-based program can preserve the beneficial impact of conventional interventions on lifestyle behaviours.

The web-based program also positively impacted the psychosocial health of participating children. In line with findings from studies examining health-related quality of life in children with overweight and obesity, participating children and their parents initially reported lower quality of life (both physical and psychosocial dimensions) regarding themselves and their children respectively, compared to generally healthy children without overweight and obesity [42, 43]. This study found significant improvements in children’s quality of life post-program completion, including children’s physical functioning which is commonly found to be impaired in children with obesity [43–45]. Quality of life scores from child and parent reports were determined to be closer to scores for generally healthy children without obesity following the web-based program [41–43]. Following the web-based program, participating children’s quality of life was found to be comparable to the quality of life of generally healthy children [43, 44, 46]. The extent of increase in children’s overall quality of life scores, determined from both child and parent reports was also clinically meaningful (4.4 or 4.5 change in overall scores regarded as meaningful differences) [43, 44, 46]. Parents in the Intervention group reported lower quality of life scores, particularly in the psychosocial dimensions, compared to the Control group at baseline. Parents’ perceptions of their children’s quality of life may relate to their knowledge of or personal experiences with the consequences of health disorders including obesity, and in turn, influence their perceived need for treatment [44, 45, 47]. Differences in quality of life scores from parent reports between cohorts at baseline, may suggest that parents in the Intervention group initially found a greater need for their child to receive the web-based program or were more motivated compared to parents in the Control group. Family motivation however, was unexplored in the primary analyses of the study.

Despite the fact that the web-based program was shorter or required less time each week to complete structured activities compared to other effective lifestyle interventions, including with varying degrees of e-Health components, clinically meaningful weight-based and quality of life outcomes were achieved [13, 48, 49]. This may be related to the high compliance/retention rate achieved during the intervention period (families completing on average 9/10 online modules and coaching sessions). Family engagement in the web-based program exceeds attendance rates reported in studies on a similar in-person group-based program, with less than 60% of families attending about 70–80% of program sessions [39, 40]. This suggests that if established as a treatment service in childhood obesity treatment plans, e-Health interventions can increase the reach of treatment, which is a limitation of effective conventional treatment services [12, 14, 18, 19]. This finding also complements evidence from another study on the potentially greater cost-effective advantage of e-Health compared to in-person interventions for treating childhood obesity [50]. In other words, theoretically, if e-Health interventions are favourably accepted by families compared to their conventional treatment counterparts, the provision of childhood obesity treatment services may at least exclude the cost of resources needed to run in-person sessions. For instance, for the web-based program, much of the education is delivered to families via digital technology (website). Education involving contact time with a healthcare professional (e.g. coaching session) is therefore, minimised compared to if the treatment service was delivered face-to-face. Further research is needed to confirm the cost-effective advantage of effective family-focused e-Health interventions and examine the factors influencing the improved reach observed in this study. Some families had multiple children included in this study (17 families) which may influence program engagement, however whether the simultaneous enrolment of siblings in a family-focused e-Health intervention encourages participation, may be a topic for further research.

Populations are disproportionally impacted by childhood obesity, with families residing in rural/regional versus metropolitan or socio-economically disadvantaged versus advantaged areas, or identifying with Aboriginal Australian/Maori Pacific Islander versus European ethnicities being at increased risk of childhood obesity [41]. These families also have less access to treatment services compared to general populations [40]. A greater proportion of children in this study were found to access the web-based program from areas with higher socio-economic advantage versus disadvantage, and with parents who reported owning their own accommodation and are employed. Participating families were more likely to be living with socio-economic advantage rather than disadvantage [29]. The proportion of children in this study found to access the web-based program from disadvantaged areas (14% of children living in decile 1,2 areas) is similar to the proportion of people in Victoria reportedly living in these areas in 2016 (16% of children living in decile 1,2 areas) [51]. The study sample may be representative of families with children living with overweight/obesity in Victoria. This may suggest that a web-based program can increase accessibility of treatment for families with children with overweight/obesity. Further research is needed to better tailor e-Health interventions for treating childhood obesity to diverse populations and maximise the benefits of effective childhood obesity treatment programs.

This is one of the few RCTs showing the effectiveness of adapting an evidence-based family-focused healthy lifestyle program to an e-Health (web-based) intervention that is entirely technology based, to families with children living with overweight/obesity. The study methodology was pragmatic in nature and delivered the program as it would usually run outside of study conditions, across Victoria. Study findings have several implications for the treatment of childhood obesity. Results show that e-Health interventions, including programs with weekly contact time with health professionals online, can be as effective, if not enhance the effectiveness of conventional treatment services, to improve weight-based and health-related behaviour change outcomes. E-Health interventions have the potential to upscale and address limitations, including poor accessibility, of current treatment services. Healthcare practitioners and program developers should consider e-Health interventions as a mode of delivering childhood obesity treatment, to help meet the increased demand for treatment from families with children with overweight/obesity.

Whereas this study supports the promising effects of a web-based program, there are several limitations to consider. Positive and clinically meaningful benefits of the web-based program found in this RCT only demonstrate the immediate impact of the e-Health intervention. This study continues to follow up with families up to 12 months post-program completion, with the intention to also evaluate the effectiveness of the web-based program during longer time points (i.e. sustained program effects). All outcome measures were self-reported by families, which may be limited by response bias. This method of data collection was chosen however, to keep in-line with the online nature of the web-based program. Allowing for the self-reporting of outcome measures is also non-invasive, a recommended method to examine subjective measures, and can increase participation, including with children, particularly when administered online [52]. Existing literature supports the accuracy of parents reporting their children’s measurements in an online study [53]. As parents supported participating children to complete study assessments, it is unclear whether children’s responses were confounded by parental influences. With an intention to deliver the web-based program as it would typically run outside of study conditions, the program was delivered during ten school terms within two years. This study did not however, account for the possibility that environmental factors, including changes in season or observance of holidays, may influence children’s health-related/lifestyle behaviours. Moreover, although this study achieved high compliance during the intervention period, it is important to note that 28 children (24 families) were lost to follow-up or withdrew post-allocation, at baseline (i.e. no baseline assessments were completed and prior to program commencement). However, all participants in the Intervention group who completed baseline measures completed at least the primary assessment at 10-weeks, following the web-based program. Only a few participants who completed baseline assessments, were non-responsive or withdrew at 10-weeks (9 children from 8 families randomised to the control group). Intention-to-treat analysis was performed on the primary outcome measure, to minimise the likelihood of attrition bias. Future research exploring families’ experiences at program registrations or leading up to program commencement would be beneficial to inform program uptake and thus, program scalability.

## Conclusions

A 10-week web-based healthy lifestyle program was effective in influencing clinically meaningful health outcomes in primary school-aged children with overweight/obesity in the short-term, supporting the use of e-Health approaches in the clinical management of childhood obesity. Results showed that e-Health interventions, including programs with weekly contact time with healthcare professionals online, can impact weight maintenance and improved diet quality, physical activity levels, and quality of life, as intended via conventional treatment services. Further research exploring factors influencing program effectiveness (e.g. research examining determinants of family engagement and compliance with treatment), evaluating the sustained impact of web-based treatment programs, and understanding ways to equitably tailor childhood obesity e-Health treatment interventions to diverse populations, is warranted to better understand the scalability of e-Health interventions for treating childhood obesity to inform health policy.

## Supplementary Information

Additional file 1: Table S1-Baseline differences in children’s dietary intake and physical activity outcome measures between groups. Baseline differences in outcome measures related to dietary intake between completers (participating children) in Intervention versus Control group.

Additional file 2: Table S2-Between group differences in change in dietary intake and physical activity outcomes over 10 weeks. Between group differences in change in secondary outcome measures related to dietary intake and physical activity from baseline to 10 weeks.

Additional file 3: Table S3-Within group differences (Control group) in dietary intake and physical from immediately pre-to post-program completion. Within group differences in outcome measures related to dietary intake and physical activity for the Control group from immediately pre-to post-program completion.

## Data Availability

Synthesised data from the current study are available from the corresponding author on reasonable request.

## Abbreviations

BMI: Body mass index
E-Health: Electronic health
BHPO: Online Better Health Program
RCT: Randomised control trial
CONSORT: Consolidated Standards of Reporting Trials
CDC: Centers for Disease Control and Prevention
BHCo: Better Health Company
SEIFA - IRSAD: Socio-Economic Indexes for Areas - Index of relative socio-economic advantage and disadvantage
REDCap: Research Electronic Data Capture
YAP: Youth Activity Profile
AES: Australian Eating Survey
ANOVA: Analysis of variance
ARFS: Australian Recommended Food Score
IQR: Interquartile range
