# Supplementary material for: The effectiveness of a 10-week family-focused e-Health healthy lifestyle program for school-aged children with overweight or obesity: a randomised control trial
Source: BMC Public Health. 2025 Jan 7;25:59. doi: 10.1186/s12889-024-21120-5 (PMC11705843; doi:10.1186/s12889-024-21120-5)
Supplement: Supplementary file 1 — Additional file 1: Table S1-Baseline differences in children’s dietary intake and physical activity outcome measures between groups. Baseline differences in outcome measures related to dietary intake between completers (participating children) in Intervention versus Control group. [file 12889_2024_21120_MOESM1_ESM.docx]

Additional file 1

Table S1. Baseline differences in children’s dietary intake and physical activity outcome measures between groups

| **Characteristic**  **Md (IQR)** | **Intervention**  **(n=58)** | **Control**  **(n=44)** | ***P*-value ^a^** |
| --- | --- | --- | --- |
| **Dietary intake ^b^** |  |  |  |
| Monounsaturated fat (%EI) | 14 (12, 15) | 14 (13,15) | 0.874 |
| Polyunsaturated fat (%EI) | 4 (4, 5) | 5 (4, 5) | 0.613 |
| Nutrient-dense/core foods |  |  |  |
| Vegetables (%EI) | 5 (3, 7) | 5 (3, 8) | 0.491 |
| Fruits (%EI) | 6 (3, 9) | 8 (5, 11) | 0.011 |
| Breads and cereals (%EI) | 21 (16, 24) | 18 (13, 23) | 0.125 |
| Milk, yoghurt, cheese (%EI) | 12 (8, 17) | 12 (8, 20) | 0.469 |
| Lean meats, fish, poultry, eggs, nuts (%EI) | 10 (7, 14) | 12 (7, 15) | 0.251 |
| Meat alternatives (%EI) | 2 (1, 3) | 2 (1, 3) | 0.915 |
| Energy-dense/non-core foods |  |  |  |
| Sweetened drinks (%EI) | 1 (0, 2) | 1 (0, 2) | 0.304 |
| Packaged snacks (%EI) | 6 (3, 9) | 5 (3, 7) | 0.626 |
| Confectionary (%EI) | 7 (3, 10) | 4 (3, 9) | 0.328 |
| Baked sweet products (%EI) | 6 (4, 10) | 6 (3, 8) | 0.481 |
| Fried take-away meals (%EI) | 13 (9, 17) | 11 (8, 18) | 0.442 |
| Fatty meats (%EI) | 2 (1, 4) | 2 (1, 3) | 0.901 |
| Diet quality score – ARFS |  |  |  |
| Vegetables (ARFS 0-21) | 10 (6, 12) | 9 (6, 14) | 0.883 |
| Fruits (ARFS 0-12) | 6 (3, 7) | 6 (4, 8) | 0.099 |
| Protein foods - meat (ARFS 0-7) | 2 (1, 2) | 2 (1, 2) | 0.429 |
| Protein foods - meat alternative (ARFS 0-6) | 2 (1, 2) | 2 (1, 2) | 0.608 |
| Grains, breads & cereals (ARFS 0-13) | 5 (4, 6) | 5 (3, 7) | 0.669 |
| Dairy foods (ARFS 0-11) | 4 (3, 5) | 5 (3, 6) | 0.536 |
| Water (ARFS 0-1) | 1 (0, 1) | 1 (0, 1) | 0.638 |
| Extras (ARFS 0-2) | 1 (1, 2) | 1 (1, 2) | 0.889 |
| **Physical activity ^c^** |  |  |  |
| Physical activity - weekday |  |  |  |
| Average total PA time (min/d) | 70.2 (46.4, 88.8) | 60.5 (46.6, 93.1) | 0.938 |
| Average PA time at school (min/d) | 17.9 (12.4, 33.2) | 19.6 (11.1, 43.5) | 0.458 |
| Average PA time at home (min/d) | 46.3 (33.2, 58.4) | 42.2 (34.1, 50.6) | 0.182 |
| Average total PA time (min/wk) | 351.1 (232.0, 444.0) | 302.2 (233.0, 465.4) | 0.938 |
| Average PA time at school (min/wk) | 89.7 (61.8, 166.1) | 98.0 (55.6, 217.56) | 0.458 |
| Average PA time at home (min/wk) | 231.5 (165.8, 292.2) | 211.2 (170.6, 253.1) | 0.182 |
| Physical activity - weekend |  |  |  |
| Average PA time (min/d) | 73.5 (56.4, 93.9) | 75.5 (62.1, 91.6) | 0.698 |
| Average PA time (min/wk) | 147.0 (112.7, 187.7) | 150.9 (124.2, 183.2) | 0.698 |

Abbreviations: Md, median; d, day; wk, week; %EI, percentage of energy intake; ARFS, Australian recommended food score; PA, physical activity; min, minutes

^a^ Mann-Whitney U Test was conducted to test for differences in outcomes between groups

^b^ 42/44 children completed Australian Eating Survey in the Control group

^c^ 41/44 children completed Youth Activity Profile in the Control group
