# Supplementary material for: The effectiveness of a 10-week family-focused e-Health healthy lifestyle program for school-aged children with overweight or obesity: a randomised control trial
Source: BMC Public Health. 2025 Jan 7;25:59. doi: 10.1186/s12889-024-21120-5 (PMC11705843; doi:10.1186/s12889-024-21120-5)
Supplement: Supplementary file 2 — Additional file 2: Table S2-Between group differences in change in dietary intake and physical activity outcomes over 10 weeks. Between group differences in change in secondary outcome measures related to dietary intake and physical activity from baseline to 10 weeks. [file 12889_2024_21120_MOESM2_ESM.docx]

Additional file 2

Table S2. Between group differences in change in dietary intake and physical activity outcomes over 10 weeks

| **Characteristic**  **Md(IQR)** | **Intervention**  **(n=58)** | | | **Control**  **(n=44)** | | | ***P-*value ^a^** |
| --- | --- | --- | --- | --- | --- | --- | --- |
|  | **Baseline** | **10 weeks** | **Change** | **Baseline** | **10 weeks** | **Change** |  |
| **Dietary intake ^b^** |  |  |  |  |  |  |  |
| Monounsaturated fat (%EI) | 14 (12, 15) | 14 (12, 15) | 0 (-2, 1) | 14 (13, 15) | 14 (13, 15) | 0 (-1, 1) | 0.729 |
| Polyunsaturated fat (%EI) | 4 (4, 5) | 5 (4, 5) | 0 (0, 1) | 5 (4, 5) | 4 (4, 5) | 0 (-1, 0) | 0.106 |
| Nutrient-dense/core foods |  |  |  |  |  |  |  |
| Vegetables (%EI) | 5 (3, 7) | 7 (5, 9) | 1 (0, 4) | 5 (3, 8) | 5 (2, 7) | 0 (-2, 1) | < 0.001 |
| Fruits (%EI) | 6 (3, 9) | 9 (6, 13) | 2 (0, 5) | 8 (5, 11) | 8 (5, 10) | 0 (-4, 1) | < 0.001 |
| Breads and cereals (%EI) | 21 (16, 24) | 21 (18, 27) | 1 (-2, 8) | 18 (13, 23) | 20 (14, 25) | 1 (-4, 5) | 0.538 |
| Milk, yoghurt, cheese (%EI) | 12 (8, 17) | 15 (10, 20) | 2 (-3, 7) | 12 (8, 20) | 14 (9, 19) | 1 (-3, 7) | 0.548 |
| Lean meats, fish, poultry, eggs, nuts (%EI) | 10 (7, 14) | 13 (9, 17) | 3 (-1, 6) | 12 (7, 15) | 11 (7, 15) | -1 (-4, 3) | 0.005 |
| Meat alternatives (%EI) | 2 (1, 3) | 3 (2, 5) | 1 (0, 3) | 2 (1, 3) | 2 (1, 3) | 0 (-1, 1) | < 0.001 |
| Energy-dense/non-core foods |  |  |  |  |  |  |  |
| Sweetened drinks (%EI) | 1 (0, 2) | 1 (0, 2) | 0 (-1, 0) | 1 (0, 2) | 1 (0, 2) | 0 (-1, 0) | 0.674 |
| Packaged snacks (%EI) | 6 (3, 9) | 3 (1, 6) | -2 (-4, 0) | 5 (3, 7) | 5 (3, 7) | 0 (-2, 2) | 0.004 |
| Confectionary (%EI) | 7 (3, 10) | 4 (2, 5) | -3 (-5, 0) | 4 (3, 9) | 7 (3, 10) | 1 (-1, 4) | < 0.001 |
| Baked sweet products (%EI) | 6 (4, 10) | 3 (2, 6) | -2 (-6, 0) | 6 (3, 8) | 6 (3, 9) | 0 (-2, 3) | 0.005 |
| Fried take-away meals (%EI) | 13 (9, 17) | 10 (8, 14) | -3 (-6, 1) | 11 (8, 18) | 11 (9, 15) | 0 (-3, 1) | 0.046 |
| Fatty meats (%EI) | 2 (1, 4) | 1 (1, 2) | 0 (-2, 0) | 2 (1, 3) | 2 (1, 3) | 0 (-1, 1) | 0.057 |
| Diet quality score – ARFS |  |  |  |  |  |  |  |
| Vegetables (ARFS 0-21) | 10 (6, 12) | 10 (8, 13) | 1 (-1, 5) | 9 (6, 14) | 10 (5, 14) | 0 (-1, 2) | 0.163 |
| Fruits (ARFS 0-12) | 6 (3, 7) | 7 (5, 8) | 1 (0, 3) | 6 (4, 8) | 6 (4, 8) | 0 (-2, 1) | < 0.001 |
| Protein foods - meat (ARFS 0-7) | 2 (1, 2) | 2 (2, 3) | 0 (0, 1) | 2 (1, 2) | 2 (1, 3) | 0 (0, 1) | 0.188 |
| Protein foods - meat alt (ARFS 0-6) | 2 (1, 2) | 2 (1, 3) | 0 (0, 1) | 2 (1, 2) | 2 (1, 2) | 0 (-1, 0) | 0.043 |
| Grains, breads & cereals (ARFS 0-13) | 5 (4, 6) | 7 (5, 8) | 1 (0, 3) | 5 (3, 7) | 5 (4, 7) | 0 (-1, 1) | 0.014 |
| Dairy foods (ARFS 0-11) | 4 (3, 5) | 5 (3, 6) | 0 (0, 1) | 5 (3, 6) | 4 (3, 5) | 0 (-1, 1) | 0.158 |
| Water (ARFS 0-1) | 1 (0, 1) | 1 (1, 1) | 0 (0, 1) | 1 (0, 1) | 1 (1, 1) | 0 (0, 0) | 0.116 |
| Extras (ARFS 0-2) | 1 (1, 2) | 1 (1, 2) | 0 (-1, 0) | 1 (1, 2) | 1 (1, 2) | 0 (0, 0) | 0.225 |
| **Physical activity ^c^** |  |  |  |  |  |  |  |
| Physical activity - weekday |  |  |  |  |  |  |  |
| Average total PA time (min/d) | 70.2 (46.4, 88.8) | 70.3 (56.9, 88.8) | 1.9 (-5.3, 10.7) | 60.5 (46.6, 93.1) | 55.6 (43.7, 94.4) | -1.7  (-11.5, 10.1) | 0.061 |
| Average PA time at school (min/d) | 17.9 (12.4, 33.2) | 17.4 (7.8, 35.5) | 0.1 (-2.4, 4.8) | 19.6 (11.1, 43.5) | 19.6 (9.0, 40.6) | 0.0 (-3.4, 0.0) | 0.066 |
| Average PA time at home (min/d) | 46.3 (33.2, 58.4) | 51.9 (41.3, 61.2) | 3.0 (-2.1, 11.3) | 42.2 (34.1, 50.6) | 40.5 (30.3, 52.9) | 0.0 (-7.4, 7.4) | 0.071 |
| Average total PA time (min/wk) | 351.1  (232.0, 444.0) | 351.3  (284.4, 444.0) | 9.5  (-26.3, 53.7) | 302.2  (233.0, 465.4) | 277.9  (218.3, 472.0) | -8.5  (-57.4, 50.7) | 0.061 |
| Average PA time at school (min/wk) | 89.7 (61.8, 166.1) | 87.2 (38.9, 177.4) | 0.37  (-11.9, 24.1) | 98.0 (55.6, 217.56) | 98.0 (45.1, 203.0) | 0.0 (-17.1, 0.0) | 0.066 |
| Average PA time at home (min/wk) | 231.5  (165.8, 292.2) | 259.4  (206.5, 305.9) | 14.8  (-10.4, 56.4) | 211.2  (170.6, 253.1) | 202.6  (151.5, 264.7) | 0.0  (-37.1, 36.9) | 0.074 |
| Physical activity - weekend |  |  |  |  |  |  |  |
| Average PA time (min/d) | 73.5 (56.4, 93.9) | 83.5 (73.3, 101.0) | 9.1  (-8.6, 18.6) | 75.5 (62.1, 91.6) | 75.1 (57.1, 92.9) | 0.0  (-18.2, 18.6) | 0.056 |
| Average PA time (min/wk) | 147.0  (112.7, 187.7) | 167.0  (146.5, 202.1) | 18.2  (-17.2, 37.1) | 150.9  (124.2, 183.2) | 150.2  (114.3, 185.9) | 0.0  (-36.5, 37.1) | 0.056 |

Abbreviations: Md, median; IQR, interquartile range; d, day; wk, week; kJ, kilojoules; %EI, percentage of energy intake; ARFS, Australian recommended food score; PA, physical activity; min, minutes

^a^ Mann-Whitney U Test was conducted to test for differences between groups

^b^ Baseline: 53/58 children completed Australian Eating Survey in the Intervention group, 42/44 children completed Australian Eating Survey in the Control group; 10 weeks: 53/58 children completed Australian Eating Survey in the Intervention group, 44/44 children completed Australian Eating Survey in the Control group; Change in outcome measures calculated for 53/58 children from the Intervention group, 42/44 children from the Control group

^c^ 54/58 children completed Youth Activity Profile in the Intervention group; 41/44 children completed Youth Activity Profile in the Control group
