# Supplementary material for: The effectiveness of a 10-week family-focused e-Health healthy lifestyle program for school-aged children with overweight or obesity: a randomised control trial
Source: BMC Public Health. 2025 Jan 7;25:59. doi: 10.1186/s12889-024-21120-5 (PMC11705843; doi:10.1186/s12889-024-21120-5)
Supplement: Supplementary file 3 — Additional file 3: Table S3-Within group differences (Control group) in dietary intake and physical from immediately pre-to post-program completion. Within group differences in outcome measures related to dietary intake and physical activity for the Control group from immediately pre-to post-program completion. [file 12889_2024_21120_MOESM3_ESM.docx]

Additional file 3

Table S3. Within group differences (Control group) in dietary intake and physical from immediately pre-to post-program completion

| **Characteristic** | **Immediately pre-program/10 weeks**  **(n=44)** | **Immediately post-program completion**  **(n=44)** | ***P*-value ^a^** |
| --- | --- | --- | --- |
| **Dietary intake, Md(IQR) ^b^** |  |  |  |
| Monounsaturated fat (%EI) | 14 (13, 15) | 14 (12, 15) | 0.762 |
| Polyunsaturated fat (%EI) | 4 (4, 5) | 4 (4, 5) | 0.468 |
| Nutrient-dense/core foods |  |  |  |
| Vegetables (%EI) | 5 (2, 7) | 8 (4, 9) | < 0.001 |
| Fruits (%EI) | 8 (5, 10) | 8 (6, 12) | 0.168 |
| Breads and cereals (%EI) | 20 (14, 25) | 23 (17, 28) | 0.037 |
| Milk, yoghurt, cheese (%EI) | 14 (9, 19) | 15 (10, 21) | 0.145 |
| Lean meats, fish, poultry, eggs, nuts (%EI) | 11 (7, 15) | 12 (8, 15) | 0.059 |
| Meat alternatives (%EI) | 2 (1, 3) | 2 (1, 4) | 0.037 |
| Energy-dense/non-core foods |  |  |  |
| Sweetened drinks (%EI) | 1 (0, 2) | 0 (0, 1) | 0.004 |
| Packaged snacks (%EI) | 5 (3, 7) | 4 (2, 5) | 0.002 |
| Confectionary (%EI) | 7 (3, 10) | 4 (3, 7) | 0.022 |
| Baked sweet products (%EI) | 6 (3, 9) | 3 (2, 6) | 0.001 |
| Fried take-away meals (%EI) | 11 (9, 15) | 10 (7, 13) | 0.003 |
| Fatty meats (%EI) | 2 (1, 3) | 1 (1, 2) | 0.014 |
| Diet quality score – ARFS |  |  |  |
| Vegetables (ARFS 0-21) | 10 (5, 14) | 14 (9, 17) | < 0.001 |
| Fruits (ARFS 0-12) | 6 (4, 8) | 8 (5, 9) | 0.005 |
| Protein foods - meat (ARFS 0-7) | 2 (1, 3) | 2 (1, 3) | 0.830 |
| Protein foods - meat alt (ARFS 0-6) | 2 (1, 2) | 2 (1, 3) | 0.009 |
| Grains, breads & cereals (ARFS 0-13) | 5 (4, 7) | 7 (5, 9) | < 0.001 |
| Dairy foods (ARFS 0-11) | 4 (3, 5) | 5 (4, 7) | < 0.001 |
| Water (ARFS 0-1) | 1 (1, 1) | 1 (1, 1) | 0.034 |
| Extras (ARFS 0-2) | 1 (1, 2) | 1 (1, 1) | 0.180 |
| **Physical activity, Md(IQR) ^c^** |  |  |  |
| Physical activity - weekday |  |  |  |
| Average total PA time (min/d) | 55.6 (43.7, 94.4) | 66.1 (53.2, 100.3) | < 0.001 |
| Average PA time at school (min/d) | 19.6 (9.0, 40.6) | 20.7 (8.0, 38.2) | 0.042 |
| Average PA time at home (min/d) | 40.5 (30.3, 52.9) | 49.1 (42.9, 55.7) | < 0.001 |
| Average total PA time (min/wk) | 277.9 (218.3, 472.0) | 330.6 (266.0, 501.7) | < 0.001 |
| Average PA time at school (min/wk) | 98.0 (45.1, 203.0) | 103.7 (39.9, 190.8) | 0.042 |
| Average PA time at home (min/wk) | 202.6 (151.5, 264.7) | 245.3 (214.6, 278.3) | < 0.001 |
| Physical activity - weekend |  |  |  |
| Average PA time (min/d) | 75.1 (57.1, 92.9) | 79.5 (65.7, 101.0) | 0.097 |
| Average PA time (min/wk) | 150.2 (114.3, 185.9) | 158.9 (131.5, 201.9) | 0.097 |

Abbreviations: Md, median; IQR, interquartile range; d, day; wk, week; kJ, kilojoules; %EI, percentage of energy intake; ARFS, Australian recommended food score; PA, physical activity; min, minutes

^a^ Wilcoxon Signed Rank test was conducted to test for changes in outcome measures from pre- to post-program completion

^b^ Analyses conducted with data from 39/44 children

^c^ Analyses conducted with data from 40/44 children
